# Supplementary figures and images for: AMPKα Subunit Ssp2 and Glycogen Synthase Kinases Gsk3/Gsk31 are involved in regulation of sterol regulatory element-binding protein (SREBP) activity in fission yeast
Source: PLoS One. 2020 Feb 13;15(2):e0228845. doi: 10.1371/journal.pone.0228845 (PMC7018046; doi:10.1371/journal.pone.0228845)

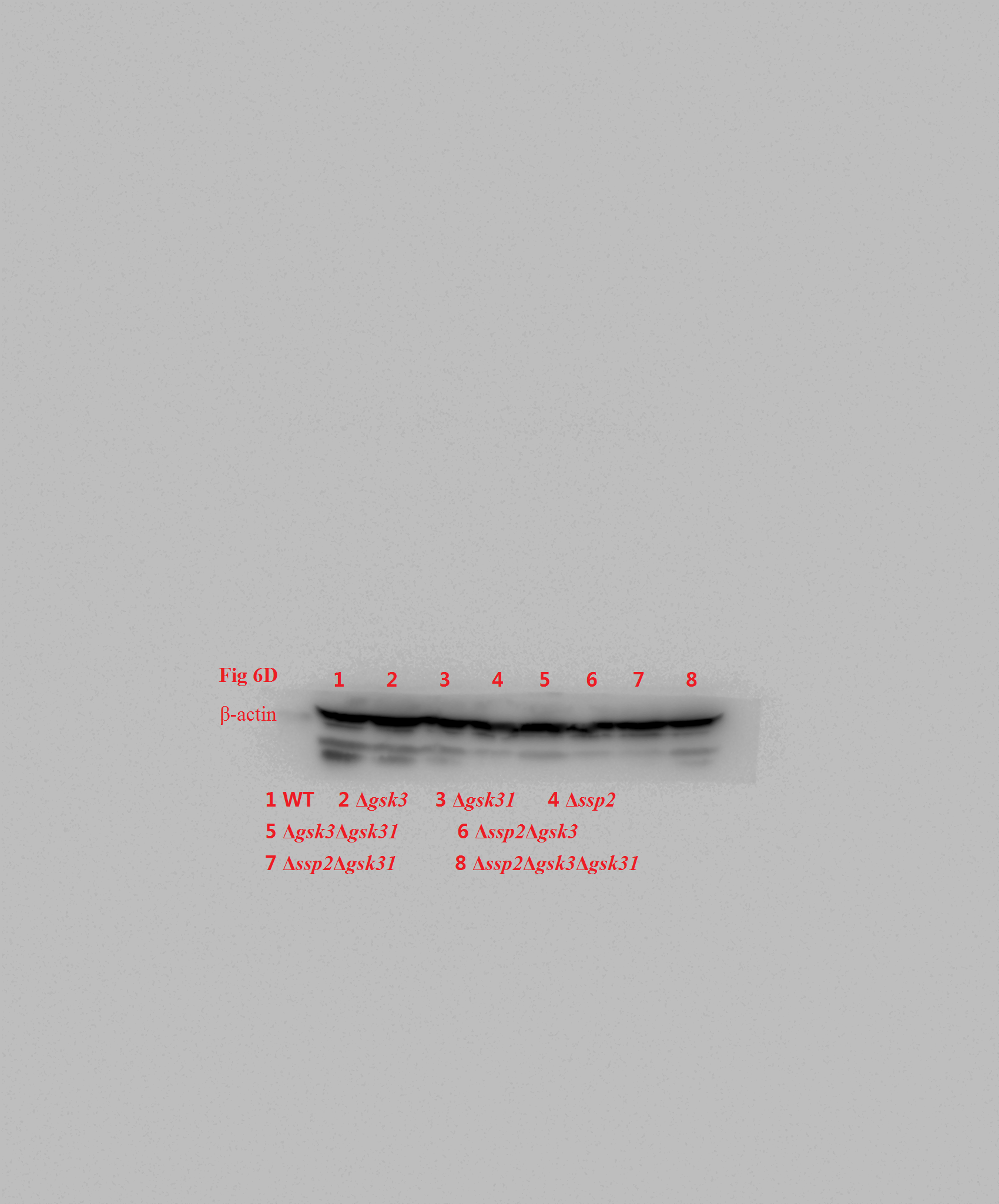

Supplement: S1 Appendix — (ZIP) [file pone.0228845.s001.zip › S1 Appendix/Fig 6D uncropped and unadjusted western blotting image-actin.bmp]

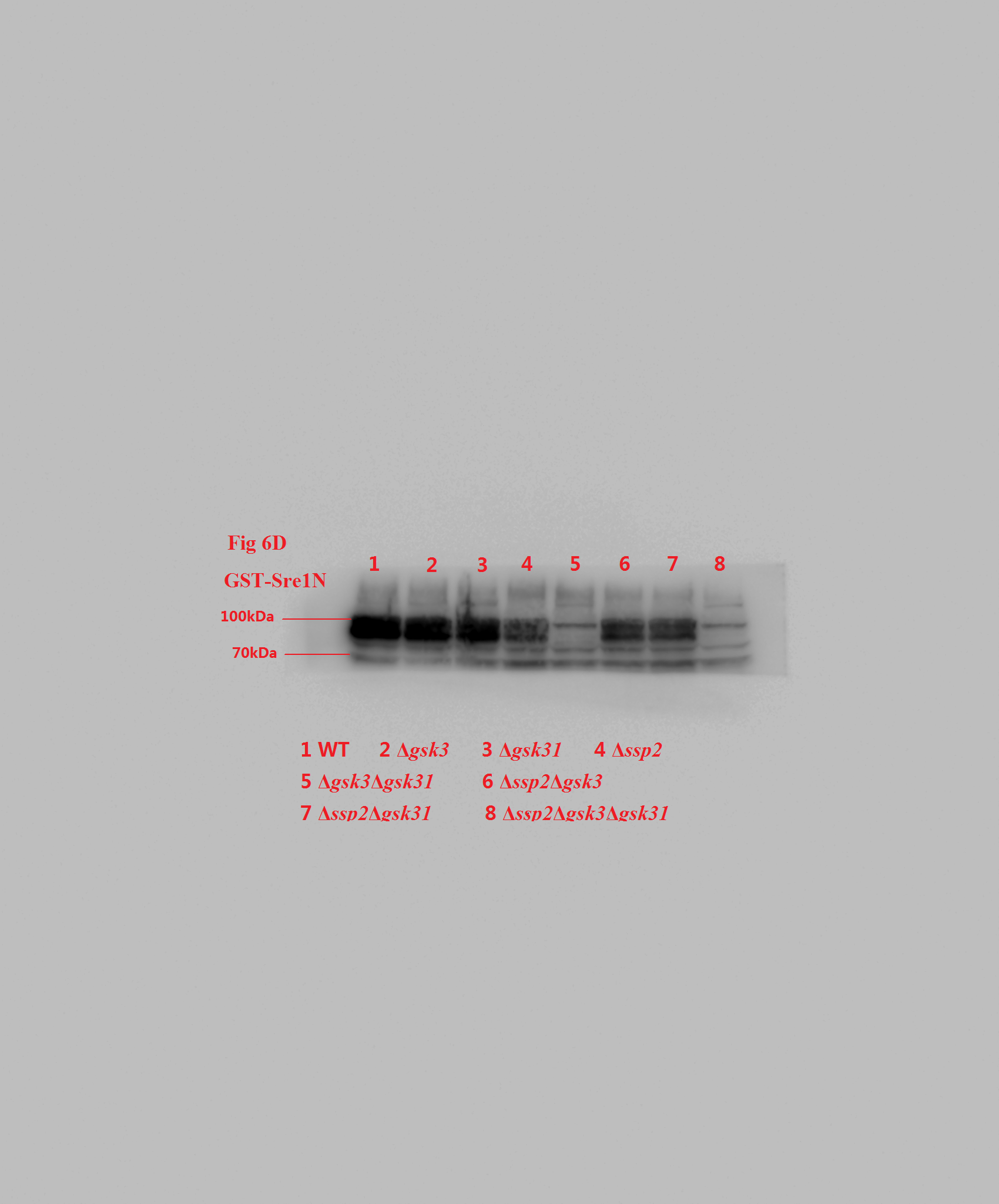

Supplement: S1 Appendix — (ZIP) [file pone.0228845.s001.zip › S1 Appendix/Fig 6D uncropped and unadjusted western blotting image-GST-Sre1N.bmp]
