# Supplementary material for: AMPKα Subunit Ssp2 and Glycogen Synthase Kinases Gsk3/Gsk31 are involved in regulation of sterol regulatory element-binding protein (SREBP) activity in fission yeast
Source: PLoS One. 2020 Feb 13;15(2):e0228845. doi: 10.1371/journal.pone.0228845 (PMC7018046; doi:10.1371/journal.pone.0228845)
Supplement: Attachment — Submitted filename: response to reviewers file.doc [file pone.0228845.s002.doc]

January 08, 2020

PLoS ONE

Dear Dr. Takashi Toda and reviewers:

Thank you for your e-mail and the reviewers' comments on our manuscript entitled “AMPKα Subunit Ssp2 and Glycogen Synthase Kinases Gsk3/Gsk31 are involved in regulation of sterol regulatory element-binding protein (SREBP) activity in fission yeast” by Hao Miao *et al*. (PONE-S-19-38064) on November 21, 2019. We would like to thank you for your very helpful comments and suggestions, and for inviting us to revise our manuscript.

In your e-mail, you’ve suggested that our revisions should address several specific points raised by two reviewers as mentioned below. Consequently, we performed a number of experiments based on the comments and were successful in obtaining new data. Thus, we address each point raised by the two reviewers.

A list of the revisions which addresses reviewers' comments is as follows:

**Reviewer #1**

**Comments**

*1. In Figure 4, they measure the luciferase activity expressed form the episomally-introduced multicopy plasmid. Although the authors conclude that the reduction of the luciferase activity in the mutant cells lacking ssp2 and/or gsk3/gsk31 is caused by deficiency of Sre1 (SREBP) function, it may be possibly caused by the reduction of the plasmid copy number. Considering that the copy number of the plasmid stably maintained cells is greatly affected by various factors, the authors cannot exclude the possibility that deletion of these genes may somehow reduce the plasmid copy number as long as the episomal plasmid is used. I strongly suggest the authors to perform the experiment using cells in which the reporter construct is integrated to the chromosome. At the minimum, they should confirm that the cells used in the experiment harbor the same number of the reporter plasmid by measuring the amount of the plasmid DNA in the cells.*

Response:

Accordingly, we constructed wild-type and Δ*sre1* chromosome-borne 3×SRE2::luc (R2.2) strains named CM150 and CM172, respectively (page 6, lines 127-131 / page 7, lines 150-153 / listed in table.1). The results showed that wild-type chromosome-borne 3×SRE2::luc (R2.2) cells could be activated by various concentration of CLZ, TER or CoCl_2_ (Fig 3A-C) in a dose-dependent manner, but in Δ*sre1* chromosome-borne 3×SRE2::luc (R2.2) cells, an extremely low response upon stimulation was observed (Fig 3D-F). These results are consistent with those obtained with multicopy reporter and suggested that both the episomal multicopy and the chromosome integration 3×SRE2::luc (R2.2) reporter could reflect Sre1 activity in living cells. These results are incorporated in the revised manuscript (page 12-13, lines 259-269 / Fig 3 and legend, page22, lines 449-462).

*2.* *In Figure 5, the authors claims that the reduction of GFP-Sre1N fluorescence in the nucleus in the mutant cells is caused by degradation of the GFP-Sre1N protein. However, I believe that it is equally possible that deletion of ssp2 and/or gsk3/gsk31 genes may somehow perturb translocation of the GFP-Sre1N protein from the cytoplasm to the nucleus and the protein may be dispersed throughout the cells. The authors should perform the immunoblotting experiment to show more directly that the whole amount of GFP-Sre1N is reduced in the mutants. If the accumulation of degraded product of GFP-Sre1N in the mutant could be detected in immunoblot, their conclusion would be further strengthened. If phosphorylation of Sre1N causes its band-shift, the author may be also able to see how much the Sre1N phosphorylation is affected by deletion of ssp2 and/or gsk3/gsk31 genes.*

Response:

We thank the reviewer for giving us this very helpful comment. Accordingly, we performed the immunoblot assay to detect the total Sre1N protein levels in wild-type and mutant cells (page 10, lines 201-214). Consistent with fluorescence intensity in wild-type cells and the mutants, the whole amount of Sre1N was markedly reduced in these mutants compared with that of wild-type cells. Notably, the whole amount of Sre1N in Δ*ssp2*Δ*gsk3*Δ*gsk31* triple deletion was lower than that in any of their single or double deletions assessed by immunoblot analysis (Fig 6D). These are incorporated in the revised manuscript (page 15-16, lines 313-314, 329-337 / Fig 6D and legend, page 24-25, lines 493-494, 507-514).

**Reviewer #2**

**Comments**

*Technical:
1. The luciferase-based reporter is introduced into cells as a plasmid, but I do not see how maintenance of the plasmid is selected. The Methods section states that transformants were cultured in ‘normal EMM media.’ Is there an auxotrophic or antibiotic-based selection to make sure that cells have andmaintain the plasmid? This is important because changes in plasmid maintenance would alter the results.*

Response:

Accordingly, there is an auxotrophic based selection for the transformants to make sure that cells have and maintain the plasmid described as follow: the multicopy 3×SRE2::luc (R2.2) reporter vector containing leucine marker can complement the *S.pombe* mutations leu1 (for example, wide-type cells HM123 (*h^-^ leu1-32*)), then the transformants would be successfully selected in EMM media without leucine. These are incorporated in the revised manuscript (page 7-8, lines 153-156).

*2. For the luciferase-based assays, is the total luciferase signal normalized to cell number? It is important to control for changes in the growth rate of cells, so the light units should be normalized to cell number in each case.*

Response:

Accordingly, for all the luciferase-based assays, we measured the cell density (OD660) of each groups at the peak time, and the luciferase light units of peak height were normalized to the corresponding cell density at the peak time. The figures and figure legends (Fig 1D-F and legend, page 21, lines 431-433 / Fig 2B and legend, page 22, lines 443-444 / Fig 3D-F and legend, page 22, lines 459-460 / Fig 4A-C and legend, page 23, lines 468-469 / Fig 5A-C and legend, page 23, lines 480-481) as well as a description of the results (page 14, lines 297-299) are modified in the revised manuscript.

*Data interpretation:
1. sre1∆ cells retain some luciferase activity upon treatment with CoCl_2_, suggesting Sre1-independent transcription of the reporter. I do not disagree with this interpretation, but the authors should note that the response is two orders of magnitude reduced from wild type cells.*

Response:

Although ∆*sre1* cells transformed with 3×SRE2::luc (R2.2) multicopy plasmid reporter retain some luciferase activity upon treatment with CoCl_2_, it is still unclear what its physiological significance is. We further constructed wild-type and Δ*sre1* chromosome-borne 3×SRE2::luc (R2.2) strains, and found that CoCl_2_-induced increase in SRE2 reporter activity was almost abolished in Δ*sre1* cells (Fig 3). Therefore, we removed the related description of Sre1-independent activation as well as partially enlarged part in Fig 1 and Fig 2. According to the reviewer’s comment, we noted that, when *sre1*^+^ was knocked out, Sre1 activity stimulated with CoCl_2_ decreased to less than 1% compared to that of wild-type cells. These are included in the revised manuscript (page 12, line 248, 254-256 / Fig 1D-F / Fig 2B and legend, page 21, line 437, 440-441).

*2. The authors measure Sre1N-GFP levels in the nucleus, and conclude that degradation has been impacted in their mutants. However, their measured differences are also consistent with altered shuttling between the nucleus and cytoplasm. They should measure total cellular levels if they want to conclude changes in total protein levels.*

Response:

Accordingly, immunoblot analysis was performed to detect the total Sre1N protein levels in wild-type cells and the mutants (page 10, lines 201-214). Consistent with their decreased fluorescence intensity of GFP-Sre1N in the nucleus, the total cellular protein levels of GST-Sre1N was also markedly reduced in the mutants. These results were included in the revised manuscript (page 15-16, lines 313-314, 329-337 / Fig 6D and legend, page 24-25, lines 493-494, 507-514).

*3. The authors could note that their results are consistent with Ssp2/Gsk3/Gsk31 acting as inhibitors of Hhp2, or alternatively acting on Sre1N activity independently of Hhp2.*

Response:

We thank this reviewer for giving us these helpful comments. Accordingly, we added some comments on these issues. These are incorporated in the revised manuscript (page 18, lines 380-382).

*4. The authors’ data seem consistent with changes in Sre1N activity as well as Sre1N protein levels. They might add this possibility into the text. In particular, they have not provided data for changes in Sre1N degradation in the new kinase mutants (e.g. lines 297-299). To make this conclusion, they would have to measure degradation rates with different assays.*

Response:

Accordingly, we added the possibility that our data are consistent with changes in Sre1N activity in the text (page15, line 328-329). To test whether the changes in Sre1N activity were consistent with Sre1N protein levels in the kinase mutants, we performed the immunoblot analysis to detect the total protein levels of Sre1N in wild-type cells and the deletion mutants. The results showed that the whole amount of GST-Sre1N was markedly reduced in the mutants except that in Δ*gsk3* or Δ*gsk31* cells. Notably, similar to the changes in Sre1N activity detected by luciferase reporter assay, the whole amount of Sre1N in Δ*ssp2*Δ*gsk3*Δ*gsk31* triple deletion was lower than that in any of their single or double deletions. These results suggested that the changes in Sre1N activity were consistent with Sre1N protein levels. These are incorporated in the revised manuscript (page17-18, lines 374-380)

We hope that these revisions would satisfy your requirements. Thank you very much for your time and consideration.

Sincerely,

Yue Fang, M.D, Ph.D.

Department of Microbial and Biochemical Pharmacy,

School of Pharmacy, China Medical University,

No.77 Puhe Road, Shenyang North New Area, Shenyang 110112, China

TEL: +86-18900910820, FAX: +86-24-31939448

Email: yfang@cmu.edu.cn
