# Supplementary material for: AMPKα Subunit Ssp2 and Glycogen Synthase Kinases Gsk3/Gsk31 are involved in regulation of sterol regulatory element-binding protein (SREBP) activity in fission yeast
Source: PLoS One. 2020 Feb 13;15(2):e0228845. doi: 10.1371/journal.pone.0228845 (PMC7018046; doi:10.1371/journal.pone.0228845)
Supplement: Attachment — Submitted filename: Response to Reviewers.doc [file pone.0228845.s003.doc]

January 24, 2020

PLoS ONE

Dear Dr. Takashi Toda:

Thank you for your e-mail of January 23, 2020 on our manuscript entitled “AMPKα Subunit Ssp2 and Glycogen Synthase Kinases Gsk3/Gsk31 are involved in regulation of sterol regulatory element-binding protein (SREBP) activity in fission yeast” by Hao Miao *et al*. (PONE-D-19-30640R1). We would like to thank you for your very helpful comments and suggestions, and for inviting us to revise our manuscript. Both reviewers raised the same point that is the interpretation of the immunoblotting data shown in Figure 6D. Accordingly, we revised our manuscript.

The revision which addresses reviewer's comment is as follows:

**Reviewer #1**

**Comment**

*The criticisms raised against the original version are properly responded, and the manuscript is satisfactorily revised. I have one minor comment regarding the interpretation of the new result of immunoblotting in Figure 6.

Minor point:
While nuclear accumulation of GFP-Sre1N appears to be diminished to a similar extent in the ssp2 mutant and the gsk3 gsk31 double mutant, the total amount of GST-Sre1N was markedly different in these two mutants (Figure 6B and D). Thus, it seems possible that Ssp2 regulates not only the degradation of Sre1N but also its translocation to the nucleus, whereas Gsks regulate mainly its degradation. It may be better to discuss this possibility.*

Response:

Accordingly, we added some comments on this issue in the revised manuscript (pages 3, lines 46-50 / page 5, lines 99-101 / page 15, line 310 / page 16, lines 334-344 / page 17, lines 353-354 / page 18, lines 379-384, 395 / page 25, lines 512-515).

**Reviewer #2**

**Comment**

*The authors have done a nice job revising the manuscript and adding new data to address the reviewer comments. I have one lingering concern related to the new immnunoblot experiment in Figure 6D. This new experiment was added in response to both reviewers, who noted that the authors should test total cellular levels of GST-Sre1N. The authors conclude that levels are decreased in a similar pattern to the nuclear levels that were determined by microscopy (e.g. page 16, lines 335-337; and stated again in the Discussion). I disagree and suggest changing the text to better reflect the actual results. The immunoblot shows a dramatic decrease in Sre1N levels in the gsk3 gsk31 double mutant cells. Deletion of ssp2 has a minor (if any) effect on these levels, andthe authors would need to quantify a better exposure to make this conclusion. This is different from the nuclear localization, where ssp2 mutant had a major effect that was similar to the gsk3 gsk31 double mutant. In comparing the gsk3 gsk31 double mutant versus the ssp2 gsk3 gsk31 triple mutant, there is no additive defect in Sre1N levels and the two strains look identical. These results suggest that Gsk3 and Gsk31 play the primary role in regulating Sre1N protein levels, whereas Ssp2 might regulate nuclear localization of Sre1N. The authors can address this comment by editing the text without additional experiments. The fact that this immunoblot does not perfectly repeat the microscopy experiment is actually quite interesting and reveals the potential for some specific roles for Ssp2 versus Gsk3/31 in regulating Sre1N. I encourage the authors to revise their interpretation to better reflect this nice result.*

Response:

Accordingly, we added these points in the revised manuscript (pages 3, lines 46-50 / page 5, lines 99-101 / page 15, line 310 / page 16, lines 334-344 / page 17, lines 353-354 / page 18, lines 379-384, 395 / page 25, lines 512-515).

We hope that these revisions would satisfy your requirements. Thank you very much for your time and consideration.

Sincerely,

Yue Fang, M.D, Ph.D.

Department of Microbial and Biochemical Pharmacy,

School of Pharmacy, China Medical University,

No.77 Puhe Road, Shenyang North New Area, Shenyang 110112, China

TEL: +86-18900910820, FAX: +86-24-31939448

Email: yfang@cmu.edu.cn
